# Infectious complications in heart transplant patients

**DOI:** 10.1186/1471-2334-14-S7-O18

**Published:** 2014-10-15

**Authors:** Brînduşa Ţilea, Nina Șincu, Simona Teches, Mihaela Ispas, Ioan Ţilea

**Affiliations:** 1 Clinical County Hospital Mureş, Romania; 2 University of Medicine and Pharmacy Tîrgu Mureş, Romania; 3 Institute of Cardiovascular Diseases and Transplantation Tîrgu Mureş, Romania; 4 Emergency Clinical County Hospital Tîrgu Mureş, Romania

## Background

Up to 75% of the patients with heart transplant present signs of an infectious episode within the first year post-heart transplant. Objectives: assessment of infections prevalence, correlation between cytomegalovirus (CMV) infection and post-transplant complications, occurrence of acute rejection and allograft vessel disease.

## Methods

37 patients who underwent orthotopic heart transplantation have been followed for a period of five years. Post-transplantation screening has been performed over the following etiological agents: CMV, hepatitis B virus, hepatitis C virus, Epstein-Barr virus and *Toxoplasma gondii*. Receptors with criteria for CMV IgG and *Toxoplasma* IgG antibodies have been accepted. Infectious episodes at 1, 6 and 12 months and evolution depending on CMV screening results, correlated with infection, acute rejection and allograft vessel disease have been pursued. Microbiological techniques, echocardiography, chest X-ray and endomyocardial biopsy have been performed for diagnosis.

## Results

The mean age of patients was 38.5 years. Within the range of 1 to 6 months, a number of 50 infectious episodes (IE) were identified, with an average rate of 1.35 IE occurrences per patient. After 6 months 68 IE were identified, with an average rate of 1.83 IE occurrences per patient. After 1 year, 22 IE were identified with an average rate of 0.59 IE occurrences per patient. Following the correlation between CMV IgG antibodies after one year and bacterial infections, 2 pulmonary infections, 4 cases of upper respiratory tract infections, 3 cases of urinary tract infections, and one sepsis case were noted. Concerning the viral infections, out of the 26 patients with CMV IgG positive antibodies 16 (61.5%) did not develop infections, 6 patients were CMV IgM antibodies positive, 3 were noted with viral pneumonia, 2 varicella zoster virus cases and one with herpes simplex virus infection were also found.

The correlation between the presence or absence of CMV IgG antibodies and rejection score has been followed. 9 CMV IgG antibody positive patients (34.6%) had the rejection score 1, 15 (57.7%) were found with rejection score 0. In CMV serology negative patients a 0 to 6 out of 11 rejection score (54.5%) was identified. The four allograft vessel diseases recorded in the study group were found in CMV IgG antibodies patients.

## Conclusion

During the evolution a decrease in survival rate related to CMV chronic infection has not been observed, but its role in the development of allograft vessel disease has been confirmed.

